# Bibliographical

**Published:** 1886-06

**Authors:** 


### BIBLIOGRAPHICAL.

THE DIAGRAM APPOINTMENT BOOK AND POCKET DIARY FOR DENTISTS. Philadelphia: Welch Dental Co., 1886.

This is one of the best forms of pocket appointment books that we have seen. There is a diagram of both the upper and lower teeth at the head of the space set apart for each day, and this may be used as a record for each filling inserted. There are three days upon each page, and thus the work for a whole week is before the eyes whenever the book is opened. It is adapted to any year. Price, with flexible leather cover, 75 cents.

THE ARCHIVES OF GYNÆCOLOGY, OBSTETRICS AND PEDIATRICS. New York City: Leonard & Co., 141 Broadway.

This is a new bi-monthly journal, especially devoted to diseases of women and children. The first number, which is before us, is a handsome one, and its one hundred and four pages are full of matter of the greatest interest to every physician. It is printed in type considerably smaller than that in which this article is set, and only "side-heads" are employed, so that every inch of space is utilized. It is, in fact, a complete compendium of gynæcological science. $3.00 per annum.

PRACTICAL NOTES ON THE TREATMENT OF SKIN DISEASES. II ECZEMA. By GEO. H. ROHE, M. D.

We have previously called attention to these exceedingly useful monographs by Dr. Rohé. The second number fully sustains the reputation gained by the first.

TRANSACTIONS OF THE ILLINOIS STATE DENTAL SOCIETY FOR 1885.

This is another of the very handsome volumes published by the S. S. White Dental Manufacturing Co. The Illinois Society is known as one of the very best (possibly we might say a little more) of all the State societies in existence. There is an earnestness and zeal exhibited by its members that might well be imitated in other States. At the meeting for 1885, ten papers and addresses were presented, some of which are of great value. The arrangement of the matter is very judicious, the report of the discussions immediately following the papers, and thus all that was said upon any subject may be found in one place and without searching through the whole book for it. The volume is a worthy successor to those which have preceded it.

PAPERS ON A SYSTEM OF REGULATING TEETH, BY INTERMITTENT PRESSURE, BASED UPON A PHYSIOLOGICAL LAW. By J. N. FARRAR, M. D., D. D. S., New York City.

Dr. Farrar has presented the most complete system for regulating teeth that has been given the profession. He has been frequently misunderstood, and his system condemned by those who did not comprehend it. We should be glad to give extracts from this series of papers in explanation of his views, but the lack of space forbids. We can only advise all dentists to make a careful study of his positive system, and can assure them that they will be much the wiser for it.

*A New Departure in Uterine Therapeutics.* The Dry Treatment. By GEO. J. ENGLEMANN, M. D. Reprint from the *St. Louis Courier of Medicine.*

*Insidious Septicæmia.* A rare, deceptive and fatal form of the disease. By GEO. J. ENGLEMANN, M. D. Reprint from Vol. IX. Gynæcological Transactions.

*Progress of Electrolysis in Surgery.* By ROBERT NEWMAN, M. D. Reprint from *Gaillard's Medical Journal.*

*What is Medicine?* Annual Address before the American Academy of Medicine. By ALBERT L. GIHON, A. M., M. D.

*Third Annual Report* of the Iowa Board of Dental Examiners.

*Cocaine in Hay Fever.* A lecture delivered at the Chicago Medical College. Reprint from the *Journal of the American Medical Association.* By SETH S. BISHOP, M. D.

*Note-Book* for cases of Ovarian and Abdominal Tumors. By JOHN HOMANS, M. D.

*Presidential Address* before the Louisiana State Dental Society. By GEO. J. FRIEDRICHS, M. D., D. D. S.

On the Necessity for the Organization of the Medical Profession. By F. E. DANIEL, M. D. Reprint from the *Medical Bulletin.*

